# *Mycobacterium* *tuberculosis* Exploits Human Interferon γ to Stimulate Macrophage Extracellular Trap Formation and Necrosis

**DOI:** 10.1093/infdis/jit097

**Published:** 2013-03-08

**Authors:** Ka-Wing Wong, Williams R. Jacobs

**Affiliations:** Department of Microbiology and Immunology, Howard Hughes Medical Institute, Albert Einstein College of Medicine, Bronx, New York

**Keywords:** *M. tuberculosis*, interferon-γ, extracellular traps, necrosis, ESX-1, human macrophages

## Abstract

Human neutrophils form extracellular traps during *M. tuberculosis* infection, but a similar phenomenon has not been reported in human macrophages. Here we demonstrate that *M. tuberculosis* induces release of extracellular traps from human macrophages. This process is regulated by elastase activity, previously shown to regulate formation of extracellular traps by neutrophils. Interestingly, formation of extracellular traps by macrophages during *M. tuberculosis* infection is inducible by interferon γ (IFN-γ). These traps are mainly produced by heavily infected macrophages. Accordingly, IFN-γ is found to stimulate *M. tuberculosis* aggregation in macrophages. Both IFN-γ–inducible events, extracellular trap formation and mycobacterial aggregation, require the ESX-1 secretion system. In addition, IFN-γ is found to enhance ESX-1–mediated macrophage necrosis. In the absence of ESX-1, IFN-γ does not restore any extracellular trap formation, mycobacterial aggregation, or macrophage necrosis. Thus, initial characterization of macrophage extracellular trap formation due to *M. tuberculosis* infection led to the uncovering of a novel role for IFN-γ in amplifying multiple effects of the mycobacterial ESX-1.

*Mycobacterium tuberculosis* is the causative agent of human tuberculosis. The World Health Organization estimates that *M. tuberculosis* was been responsible for approximately 1.5 million deaths and approximately 8.8 million new infections in 2010 [1]. A key feature of tuberculosis is the presence of necrotic caseations in the lungs, which are mainly derived from the necrotic corpses of macrophages [2]. Induction of macrophage necrosis is a well-known virulence mechanism of *M. tuberculosis* and is dependent on the ESX-1/RD1 protein secretion system [3, 4].

The ESX-1 secretion system comprises 11 gene products. ESAT-6 and CFP-10 are 2 major ESX-1 substrates. They are required for the proper functioning of the secretion system [5, 6]. ESX-1's necrotic effect has been attributed to the membrane-perforating activity of ESAT-6 [4, 7]. This ESX-1 activity is responsible for the ESX-1–dependent phagosomal rupture [8–10] and the subsequent escape of *M. tuberculosis* [11]. ESAT-6 can interact indirectly with the cell-surface innate immune receptor Toll-like receptor 2 (TLR2), causing suppression of proinflammatory cytokine responses [12].

Neutrophils undergo a novel death mechanism when subjected to infections or chemical stimulations [13]. The mechanism is characterized by the release of cellular DNA, which is decorated with citrullinated histones [14]. Initial work on PMA-stimulated neutrophils indicates that cell lysis, as measured by lactate dehydrogenase release, occurs after DNA release has been completed [15]. But a single-cell assay for lysis reveals that extracellular DNA is detected as soon as the plasma membrane is ruptured [13]. Rather than a passive leakage from cellular lysis, the DNA release is regulated by multiple enzymes, such as neutrophil elastase [16]. The released DNA is called an extracellular trap because of its ability to bind diverse pathogens ranging from yeast to bacteria [15]. Extracellular traps have reported antibacterial activities [17]. However, this model has been under scrutiny [18]. A new report indicates that bacteria released from extracellular traps can resume growth [19]. Thus, the antibacterial effect of extracellular traps should be determined in situ. Macrophages can produce extracellular traps under certain circumstance [20]. However, despite extensive studies on bacteria-induced macrophage deaths, it remains unclear whether death-inducing pathogens, such as *M. tuberculosis*, can induce human macrophages to produce extracellular traps.

Formation of extracellular traps by neutrophils upon stimulation with complement factor 5a requires priming by interferon γ (IFN-γ) [21]. Murine macrophage studies clearly show that IFN-γ induces *M. tuberculosis* killing [22, 23]. But a similar effect of IFN-γ in human macrophages remains controversial [24–26]. IFN-γ can even enhance *M. tuberculosis* replication during human macrophage infections [26]. Vogt and Nathan have recently reported specific culture conditions for human macrophages, such as physiological O_2_ levels and the presence of the growth factor granulocyte macrophage colony-stimulating factor (GM-CSF), that are critical for the IFN-γ–mediated antimycobacterial activity [27].

Here we show that *M. tuberculosis* induced extracellular trap formation by infected human macrophages. This phenomenon was mediated by elastase activity. We also demonstrated that IFN-γ amplified extracellular trap formation in an ESX-1–dependent manner. As extracellular trap formation is linked to cell death, the effect of IFN-γ on ESX-1's necrotic effect was studied. Our present results suggest a novel role for IFN-γ in amplifying multiple effects of the mycobacterial ESX-1.

## METHODS

### Preparation of Primary Human Macrophages

Peripheral blood monocytes were isolated from human peripheral blood (New York Blood Center) by density centrifugation on Ficoll-Paque PLUS (GE Healthcare), followed by positive selection, using CD14 magnetic beads (Miltenyi Biotec). CD14^+^ monocytes on the magnetic column were washed 3 times in phosphate-buffered saline (pH 7.2), 0.5% fetal bovine serum, and 2 mM ethylenediaminetetraacetic acid before elution by gravity. Selected CD14^+^ monocytes were resuspended in Roswell Park Memorial Institute (RPMI) 1640 (catalog no. 11875, Gibco-Invitrogen) containing 10% non–heat-inactivated human AB serum (Gemini Bio-Products) and 20 mM HEPES buffer with or without recombinant human GM-CSF (2 ng/mL) or macrophage colony-stimulating factor (M-CSF; 10 ng/mL; R&D Systems). Cell density was adjusted to 0.67 × 10^6^ cells/mL. A total of 0.3 or 0.6 mL of cells was dispensed respectively into 48-well or 24-well tissue culture plate wells, the latter containing coverslips. Monocytes were differentiated into macrophages for 11–16 days at 37°C in a humidified chamber with 5% CO_2_ or in an O_2_-regulated humidified chamber flushed with N_2_ to achieve 10% O_2_ along with 5% CO_2_. Thirty percent of incubating medium was replaced every 3–4 days with fresh medium with or without the corresponding growth factor. Macrophages were activated by recombinant human IFN-γ at 0, 2.5, 25, or 100 U/mL (EMD-Calbiochem) overnight before infection.

### *M. tuberculosis* Infection

*M. tuberculosis* H37Rv, Δ*ESX-1* (or Δ*RD1*), and complemented Δ*ESX-1* have been described elsewhere [4] and were grown at 37°C in Middlebrook 7H9 (Difco) supplemented with 10% oleic acid-albumin-dextrose-catalase (BBL, Becton Dickinson), 0.5% glycerol, and 0.05% Tween-80. A total of 30 µg/mL kanamycin was added to 7H9 for the complemented strain. Bacteria were prepared for infection as described elsewhere [10]. Because the optical density of nonsonicated bacteria was typically twice that of sonicated bacteria, multiplicities of infection (MOIs) of 5 for nonsonicated bacteria and 10 for sonicated bacteria were used. Four hours after infection with *M. tuberculosis*, macrophages were washed twice with RPMI 1640 containing 20 mM HEPES buffer and then maintained in RPMI 1640 containing 10% non–heat-inactivated human AB serum and 20 mM HEPES buffer. Macrophages activated by IFN-γ were incubated in the same dose of IFN-γ after infection. When appropriate, 1,25-dihydroxyvitamin D (Enzo Life Sciences) was used at 20 nM along with IFN-γ as described elsewhere [28].

### Analyses of Infected Macrophages

Necrotic macrophages were stained by 2.5 µg/mL propidium iodide (Sigma) in serum-free RPMI 1640 for 10 minutes at room temperature and fixed in 2% paraformaldehyde (Sigma) in RPMI 1640 for 1 hour. To stain *M. tuberculosis*, auramine-rhodamine staining was performed using the TB Fluorescent Stain Kit T (Becton Dickinson) according to manufacturer's protocol. To visualize extracellular traps, fixed cells were stained with Hoechst 33 258 (Sigma) or picogreen (Invitrogen-Life Technologies) for 5 minutes at room temperature. Processed cells were analyzed using Eclipse Ti (Nikon) or Axio Observer (Carl Zeiss) inverted fluorescence microscopes equipped with a Photometrics CoolSNAP HQ^2^ (Photometrics) or AxioCam Mrm (Carl Zeiss) charge-coupled device camera, respectively. Images were analyzed by ImageJ software. *M. tuberculosis* was defined as aggregate positive when rhodamine's intensity was >125 arbitrary units, based on the 0–255 scale of 8-bit images. Average sizes of the *M. tuberculosis*–positive aggregates were determined by ImageJ's Analyze Particle function. Cells were identified as necrotic when the intensity of propidium iodide was >45 arbitrary units on the 0–255 scale. Cells were identified as apoptotic when propidium iodide's intensity was <45 arbitrary units and Hoechst's intensity was >125 arbitrary units. The percentage of positive cells was defined as the number of cells with positive signals relative to the total number of cells. Data were displayed as the mean percentage obtained from 4 different fields; 400–900 cells were examined from each field.

Mycobacterial growth in macrophages was enumerated by lysis in 0.5% Triton X100, followed by spotting serial dilutions on agar (7H10, 10% OADC) in triplicate. Colonies were counted after 2–3 weeks.

### Statistical Analysis

Results were tested statistically by an unpaired 2-tailed Student *t* test in Microsoft Excel.

## RESULTS

### *M. tuberculosis* Induces Release of Extracellular Traps From Human Macrophages

**Figure 1.**
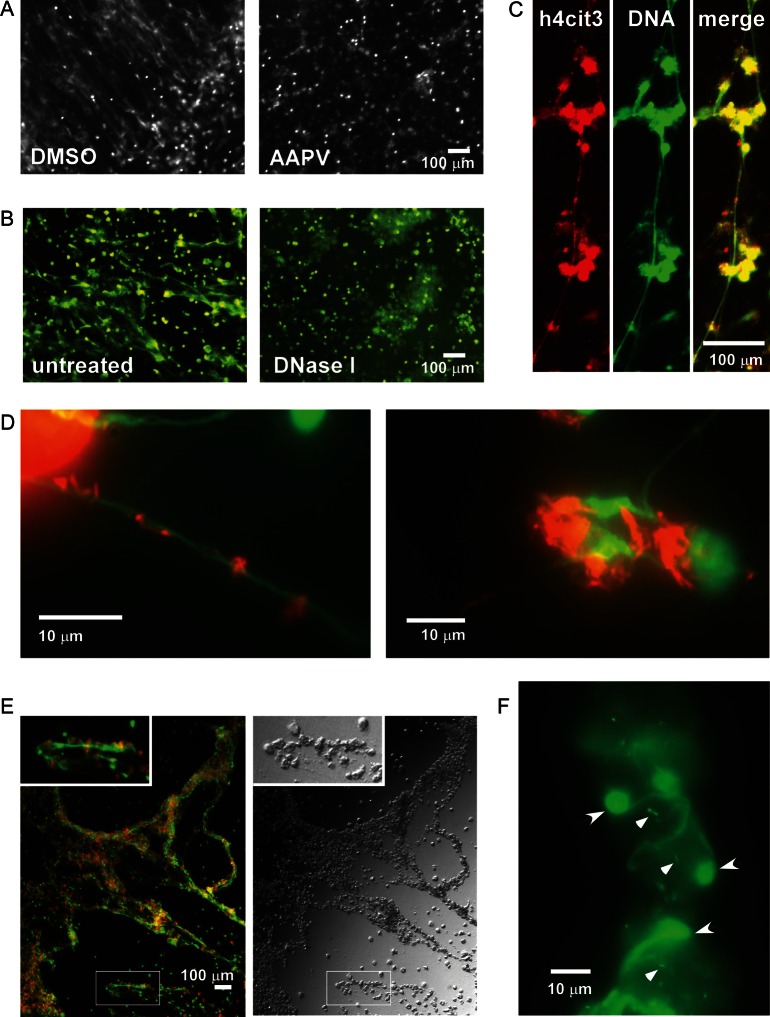
Extracellular trap formation by *Mycobacterium tuberculosis*–infected macrophages. Primary human macrophages were infected with *M. tuberculosis* H37Rv at a multiplicity of infection (MOI) of 5 without sonication of *M. tuberculosis*. *A*, Effect of elastase inhibitor AAPV. Macrophages differentiated in macrophage colony-stimulating factor (M-CSF) were pretreated 1 hour before and maintained after *M. tuberculosis* infection with either dimethyl sulfoxide (DMSO) or 100 nM AAPV. Twenty-seven hours after infection, cells were fixed and stained with Hoechst 258. *B*–*F*, Differentiation, activation, and infection of macrophages were performed in 10% O_2_. *M. tuberculosis* was not sonicated. Macrophages that were differentiated with granulocyte-macrophage colony-stimulating factor (GM-CSF) and activated with 100 U/mL of interferon γ were fixed 2 days after infection. Fixed cells were treated with DNase I (*B*) or processed for citrullinated histone 4 staining (*C*) or auramine-rhodamine staining to visualize *M. tuberculosis* (*D* and *E*), before staining with the double-stranded DNA–specific dye picogreen. The circles stained with picogreen represent intact nuclei. *F*, Nucleic origin of extracellular traps. GM-CSF–differentiated macrophages were infected with sonicated *M. tuberculosis* at an MOI of 10 and stained with picogreen. Arrows indicate nuclei that are releasing chromosome DNA. Arrowheads indicate *M. tuberculosis*.

Our preliminary observations indicated that *M. tuberculosis* infection induced PMA-differentiated THP-1 macrophages to produce Hoechst-positive fiber structures (data not shown). A similar phenomenon was also observed in human macrophages derived from peripheral blood mononuclear cells from healthy donors (Figure 1*A*). In this report, primary human macrophages from healthy donors were used throughout this study. The observed extracellular structures were morphologically identical to that of extracellular traps, which are produced by activated neutrophils and are composed mainly of DNA decorated with citrullinated histones. Staining with picogreen, a dye specific for double-stranded DNA, and DNase I treatment confirmed that the fiber structures contained DNA (Figure 1*B*). An antibody specific to histone h4 citrullinated at residue 3 also recognized the DNA fiber produced by *M. tuberculosis*–infected macrophages (Figure 1*C*).

Activated neutrophils undergo a novel cell death mechanism that leads to extracellular trap formation. Multiple enzymes, such as neutrophil elastase, regulate extracellular trap formation [16]. As macrophages also express various elastase activities [29, 30], we determined whether elastase activity affected extracellular trap formation in *M. tuberculosis–*infected macrophages. Indeed, the elastase inhibitor AAPV prevented primary human macrophages infected with *M. tuberculosis* from forming DNA fibers (Figure 1*A*). AAPV did not block macrophage necrosis triggered by *M. tuberculosis* (data not shown), indicating that DNA fiber formation was a regulated process, rather than a passive event due to cell lysis.

Acid-fast staining revealed that extracellular *M. tuberculosis* colocalized with the DNA fibers after extensive washings, indicating that *M. tuberculosis* was bound to the DNA fibers (Figure 1*D*). Thus, we characterized the DNA fibers released from infected human macrophages as extracellular traps. Mouse macrophages derived from bone marrow or of cell-line origin did not produce extracellular traps as a result of *M. tuberculosis* infection, even after onset of necrosis (data not shown).

In contrast to the extracellular mycobacteria that bind to extracellular traps, the majority of *M. tuberculosis* cells remained associated within macrophages, as expected (Figure 1*D*). As infections progressed, macrophages with a high burden of *M. tuberculosis* tended to cluster together with extracellular traps (Figure 1*E*). Higher-magnification imaging of these structures revealed that extracellular traps emerge from the nuclei of infected macrophages (Figure 1*F*). Some extracellular traps from multiple nuclei appeared to merge together in extracellular space.

Given that high mycobacterial burden was associated with extracellular trap formation in macrophages, we tested whether mycobacterial clumps were more efficient at promoting extracellular trap formation. Without sonication, mycobacterial clumps typically contained 5–20 mycobacteria. Given that nonsonicated bacteria induced the death of many more cells (data not shown), we used a lower MOI to normalize the cytotoxic effect. Generally, nonsonicated bacteria induced more extracellular trap formation than sonicated bacteria (data not shown).

### Human IFN-γ Promotes the Formation of Extracellular Traps by *M. Tuberculosis*–Infected Macrophages

**Figure 2.**
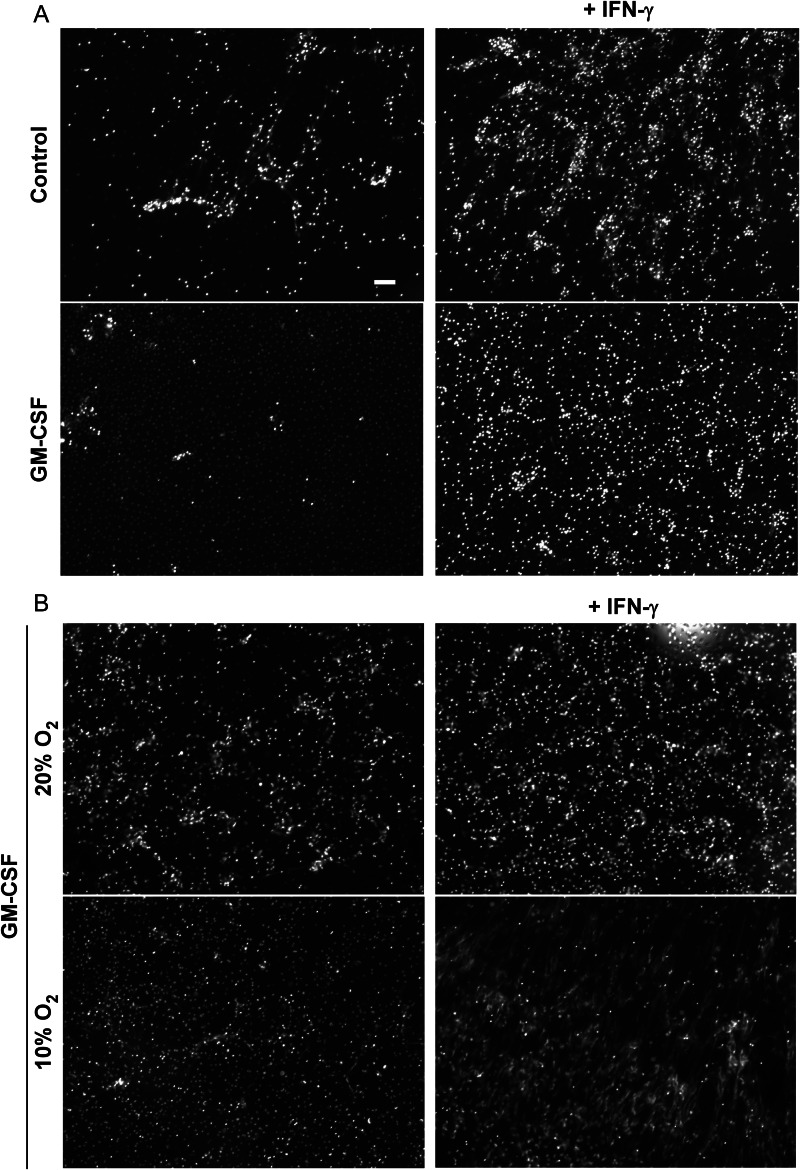
Interferon γ (IFN-γ) enhances macrophages to form extracellular traps. Hoechst staining reveals fine fiber structures that have emerged from nuclei that are morphologically indistinguishable from extracellular traps. *A*, The effect of granulocyte-macrophage colony-stimulating factor (GM-CSF) differentiation and IFN-γ activation is shown. Primary human macrophages differentiated with or without GM-CSF in the presence of 10% human serum were activated with 100 U/mL IFN-γ or left untreated before infection with sonicated *M. tuberculosis* H37Rv at a multiplicity of infection (MOI) of 10. *B,* The effect of O_2_ levels in shown. Primary human macrophages were differentiated with GM-CSF either at normoxia (approximately 20% O_2_) or at a physiological O_2_ level (10% O_2_) and pretreated with 100 U/mL IFN-γ before infection with nonsonicated *M. tuberculosis* at an MOI of 5. The corresponding phase contrast images are shown in Supplementary Figure 1. Scale bar, 100 µm.

Neutrophils stimulated with lipopolysaccharide or complement factor 5a release extracellular traps only if the neutrophils have been primed with GM-CSF [31]. In contrast to neutrophils, GM-CSF–differentiated macrophages were less efficient at forming extracellular traps in response to challenge with nonsonicated *M. tuberculosis* (Figure 2*A*). GM-CSF represses IFN-γ signaling, and priming of IFN-γ is critical for mature neutrophils to form extracellular traps in response to stimulation [21]. We therefore tested IFN-γ's effect on *M. tuberculosis*–induced extracellular trap formation. IFN-γ was found to enhance *M. tuberculosis–*induced extracellular trap formation by macrophages differentiated with M-CSF but not with GM-CSF (Figure 2*A*).

The preceding observation was made under atmospheric O_2_ levels (approximately 20%). However, macrophages are normally exposed to physiological O_2_ levels (5%–10%) [32]. Since the O_2_ level has a major impact on the metabolism and behavior of cultured human macrophages [33], we studied the effect of 10% O_2_ on extracellular trap formation in the presence or absence of IFN-γ. GM-CSF–differentiated macrophages cultured in 10% O_2_ produced no extracellular traps upon infection by *M. tuberculosis*, even when the bacteria were nonsonicated (Figure 2*B*). Remarkably, IFN-γ enabled the infected macrophages cultured in 10% O_2_ to release extracellular traps (Figure 2*B*). Under this condition, nearly all infected macrophages formed extracellular traps (Figures 1*E* and 2*B*). Thus, IFN-γ remained capable of inducing extracellular traps from macrophages regardless of whether they were cultured in approximately 20% or 10% O_2_.

### The Effect of *M. tuberculosis* ESX-1 on Extracellular Trap Formation Is Further Enhanced by Human IFN-γ

**Figure 3.**
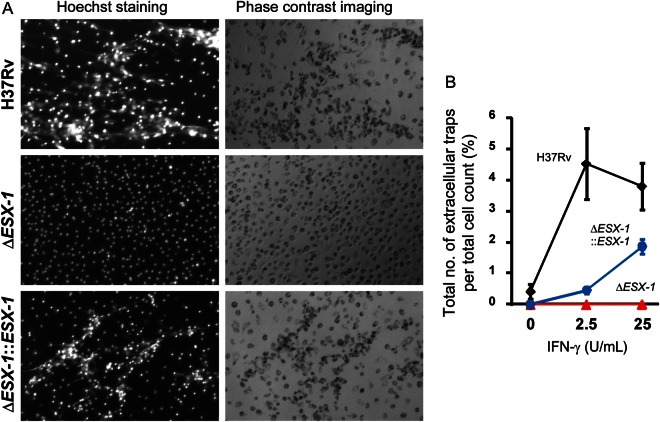
ESX-1 mediates extracellular trap formation by *Mycobacterium tuberculosis­*–infected macrophages. *A*, Primary human macrophages were infected with nonsonicated *M. tuberculosis* H37Rv at a multiplicity of infection (MOI) of 5. Hoechst staining of macrophage colony-stimulating factor–differentiated macrophages infected with *M. tuberculosis*, Δ*ESX-1*, or Δ*ESX-1*::*ESX-1* was performed 2 days after infection. *B*, Primary human macrophages differentiated with granulocyte-macrophage colony-stimulating factor in 10% O_2_ were activated with 0, 2.5, or 25 U/mL interferon γ (IFN-γ). Macrophages were analyzed 2 days after infection with sonicated H37Rv at an MOI of 10. Extracellular traps were stained by picogreen. Total numbers of extracellular traps per total cell counts were quantitated from 4 different fields.

We next examined whether ESX-1 played a role in *M. tuberculosis–*induced extracellular trap formation, since ESX-1 is required for initiating caspase-1–independent cell death in human macrophages [10, 34] and because extracellular trap formation was unaffected by caspase-1 inhibition (data not shown). An *M. tuberculosis* ESX-1 deletion mutant did not induce M-CSF–differentiated macrophages to form extracellular traps (Figure 3*A*). Addition of human IFN-γ could not restore the ability of the ESX-1 mutant to induce extracellular trap formation by GM-CSF–differentiated macrophages (Figure 3*B*). These results indicated that human IFN-γ–potentiated extracellular trap production was dependent on ESX-1. Regardless of whether bacteria were sonicated, extracellular trap formation induced by *M. tuberculosis* remained strictly dependent on the ESX-1 and was highly inducible by IFN-γ (data now shown).

### Human IFN-γ Enhances ESX-1–Dependent *M. tuberculosis* Aggregation and Growth in Macrophages

**Figure 4.**
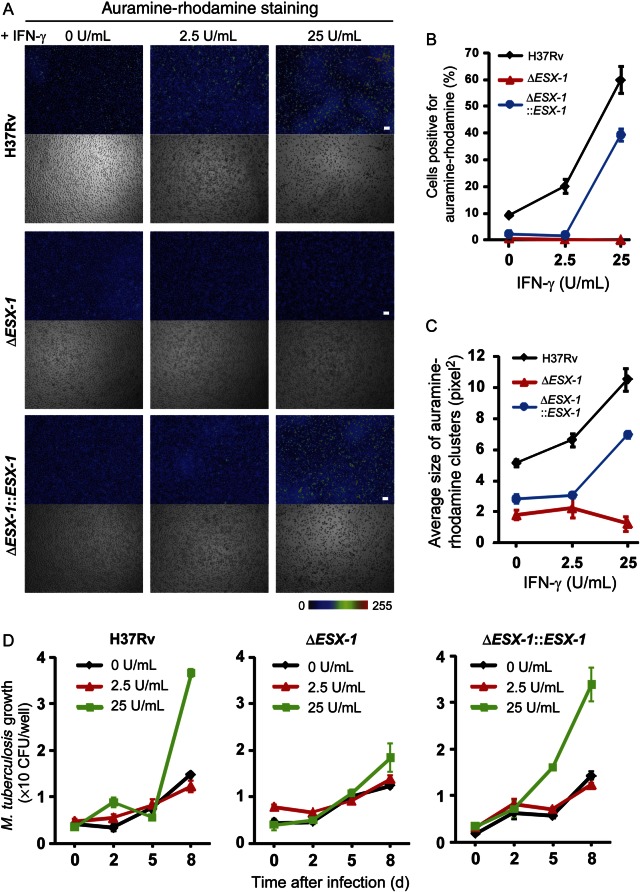
Interferon γ (IFN-γ)–enhanced *Mycobacterium tuberculosis* aggregation requires ESX-1**.** Primary human macrophages were differentiated with granulocyte-macrophage colony-stimulating factor (GM-CSF) at 10% O_2_ and were activated with the indicated dose of IFN-γ or left untreated. Macrophages were infected with sonicated H37Rv at a multiplicity of infection of 10. *A*, Auramine-rhodamine staining of fixed macrophages was performed 3 days after infection. The intensity of the rhodamine was displayed in a rainbow color scale. Scale bars, 100 µm. *B* and *C*, Results of quantification of the percentages of *M. tuberculosis* cells that were auramine-rhodamine positive (>125 on a rainbow color scale; *B*) and the average sizes of the *M. tuberculosis* aggregates (*C*) are shown. *D*, A role for ESX-1 on *M. tuberculosis* growth in the presence of different doses of IFN-γ is shown. Error bars indicate standard errors of the mean. Abbreviation: CFU, colony-forming units.

Our previous observation that extracellular traps were usually produced from heavily infected macrophages prompted us to examine whether IFN-γ would increase the number of heavily infected macrophages. Acid-fast staining of *M. tuberculosis* showed that IFN-γ induced *M. tuberculosis* aggregations in human macrophages (Figure 4*A*). Vogt and Nathan reported that IFN-γ levels of >25 U/mL are cytotoxic to mycobacteria-infected human macrophages, whereas a low IFN-γ concentration (2.5 U/mL) conferred some degree of protection to human macrophages [27]. In our case, a lower IFN-γ level (ie, 2.5 U/mL) still stimulated *M. tuberculosis* aggregation (Figure 4*A*).

Because extracellular trap formation was linked to increased *M. tuberculosis* burden and because IFN-γ enhanced the ESX-1–dependent extracellular trap formation, we tested whether the IFN-γ–enhanced *M. tuberculosis* aggregation was dependent on ESX-1. Auramine-rhodamine staining revealed that increasing IFN-γ concentrations yielded a dose-dependent increase in the size and number of *M. tuberculosis* aggregates in a strictly ESX-1–dependent fashion (Figure 4*A*–*C*). These increases coincided with the ESX-1–dependent growth enhancement in the presence of a high dose (25 U/mL) of IFN-γ (Figure 4*D*).

### Human IFN-γ Potentiates Macrophage Necrosis Induced by *M. tuberculosis* ESX-1

**Figure 5.**
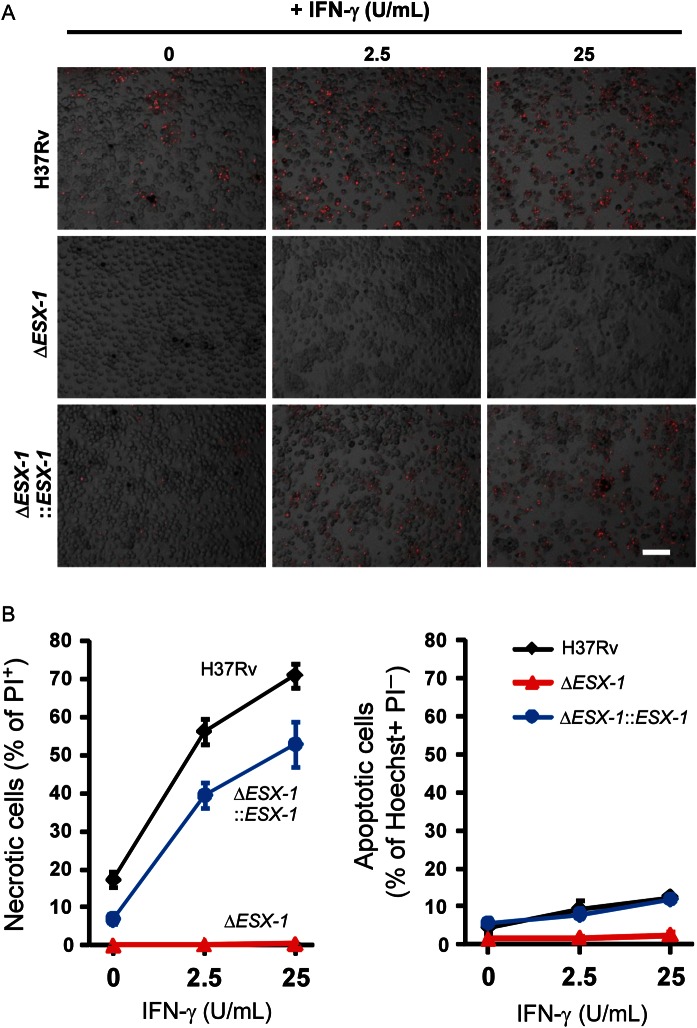
Interferon γ (IFN-γ) promotes ESX-1–triggered necrosis. Primary human macrophages differentiated with granulocyte-macrophage colony-stimulating factor (GM-CSF) in 10% O_2_ were activated with 0, 2.5, or 25 U/mL IFN-γ. Macrophages were analyzed 2 days after infection with sonicated H37Rv at a multiplicity of infection of 10. Necrotic cells were stained by propidium iodine (red) before fixation. Fixed cells were then stained with Hoechst stain for condensed nuclei as a marker for apoptotic cells (not shown for clarity). *A*, Necrotic (PI+) human macrophages are shown. Scale bar, 100 µm. *B* and *C*, Results of quantification of the frequency of necrotic (PI+; *B*) and apoptotic (Hoechst+ PI−; *C*) cells are shown. Error bars indicate standard errors of the mean. Images were quantified by ImageJ as described in Materials and Methods.

Finally, since extracellular trap formation was linked to *M. tuberculosis­–*induced necrosis, which in turn was promoted by a higher MOI, we suspected that IFN-γ would also promote the ESX-1–triggered necrosis in human macrophages. Indeed, IFN-γ pretreatment readily induced rapid necrotic death in macrophages infected with *M. tuberculosis,* whereas the same treatment had a minimal effect on macrophages infected with the ESX-1 mutant of *M. tuberculosis* (Figure 5). An increase in ESX-1–dependent extracellular trap formation due to increasing IFN-γ doses was also detected (Figure 5). Similar results were obtained from PMA-differentiated THP-1 human macrophages (data not shown). The absence of necrosis induction and extracellular trap formation by the ESX-1 deletion mutant was unlikely due to the mutant's survival defect within macrophages, because the ESX-1 deletion mutant survived as well as the wild-type when necrosis and extracellular trap formation were being quantified (Figure 4*D*). Thus, IFN-γ amplified ESX-1's multiple effects on inducing mycobacterial aggregation, extracellular trap formation, and macrophage necrosis.

## DISCUSSION

Our present study revealed a novel aspect of macrophage death induced by *M. tuberculosis*: formation of DNA-containing extracellular traps from infected human macrophages. This phenomenon took place after *M. tuberculosis* resided within macrophages, which contrasts with neutrophil extracellular trap formation triggered by extracellular bacterial pathogens. Only a subset of *M. tuberculosis* was bound to the extracellular traps, while the majority remained within the macrophages. The extent to which extracellular traps were formed increased over time as *M. tuberculosis* continued to replicate intracellularly, indicating that extracellular traps did not affect intracellular mycobacterial growth Neutrophil extracellular traps do not affect the viability of *M. tuberculosis* [35]. Whether *M. tuberculosis* in direct contact with macrophage extracellular traps is similarly unaffected has not been addressed, but our preliminary results indicate that extracellular *M. tuberculosis* in contact with extracellular traps retained cell integrity, based on propidium iodide staining (data not shown).

ESX-1 is a major virulence factor for mycobacterial pathogenesis, encoding a specialized protein secretion system and triggering a caspase-1–independent cell death pathway. We report here that ESX-1 was critical for extracellular trap formation by human macrophages. Our result stood in contrast to a recent report based on mouse macrophages, which shows that *M. tuberculosis* at a high MOI induces a small degree of DNA-containing fiber structures independent of ESX-1 [36]. We failed to detect any extracellular trap formation by mouse bone marrow–derived macrophages infected by *M. tuberculosis* at an MOI of 10, suggesting that *M. tuberculosis*–induced extracellular trap formation was a phenotype specific to human macrophages.

Observations from previous animal studies are suggestive of the existence of extracellular traps induced by *M. tuberculosis* infection. Extracellular traps can be induced in vivo by trehalose dimycolate, a cell wall component of *M. tuberculosis* in fibrinogen-deficient mice [37]. Extracellular traps are known to promote thrombosis in vitro and in vivo [38–40]. The presence of vascular thrombosis in necrotic lesions of *M. tuberculosis*–infected rabbits is consistent with the existence of extracellular traps induced by *M. tuberculosis* [41]. Conversely, the absence of vascular thrombosis is in line with our inability to detect *M. tuberculosis­*–infected murine macrophages to form extracellular traps.

We unexpectedly observed that IFN-γ stimulated extracellular trap formation by macrophages cultured at 10% O_2_ with GM-CSF. IFN-γ is a well-known macrophage activation factor that stimulates antimycobacterial activities in murine macrophages [22, 42, 43]. Its protective role against *M. tuberculosis* has been established in animal infection studies [23, 44] and inhalation treatment for patients with active tuberculosis [45]. Consistent with our observed correlation between extracellular trap formation and the intracellular burden of macrophages, we demonstrated on the basis of acid-fast staining that IFN-γ increased *M. tuberculosis* aggregation. We also detected a significant ESX-1–dependent increase in mycobacterial growth stimulated by IFN-γ at a late time point (Figure 4*D*). It is likely that the ability of IFN-γ to induce further extracellular trap formation and necrosis via an ESX-1 mechanism was mediated, at least in part, by the ability of IFN-γ to stimulate ESX-1–dependent mycobacterial aggregation and growth.

Our results collectively suggest that the mycobacterial ESX-1 secretion system subverts the IFN-γ responses of human macrophages, resulting in amplification of the pathological effects of ESX-1. To the best of our knowledge, the synergistic effect of IFN-γ and ESX-1 has not been reported. We demonstrated that ESX-1 was required for IFN-γ to promote extracellular trap formation by infected macrophages. Since necrosis induction is a primary virulence mechanism of ESX-1, the effect of IFN-γ on the ESX-1–induced necrosis was also examined. We found that IFN-γ synergized with ESX-1 to induce necrosis (Figure 3). IFN-γ is known to enhance cytotoxicity in *M. tuberculosis*–infected human macrophages [24, 36]. One report showed that IFN-γ enhanced necrosis in bacille Calmette-Guérin–infected macrophages, implying that ESX-1 was not involved [36]. Our work by contrast used the isogenic ESX-1 mutant of *M. tuberculosis* to address unequivocally the role for ESX-1.

It remains unclear how IFN-γ interacts with ESX-1 and enhances ESX-1's cellular effects. Among the synergistic effects of IFN-γ and ESX-1, necrosis occurred earlier than *M. tuberculosis* aggregation and extracellular trap formation (data not shown). Macrophages undergoing necrosis might release, actively or passively, intercellular signaling molecules that promote macrophage-macrophage interactions. Such cellular aggregation might give rise to the observed aggregation of *M. tuberculosis* and could also contribute to extracellular trap formation because of a relative higher *M. tuberculosis* burden. The mechanism through which the ESX-1–dependent necrosis is amplified by IFN-γ is unknown. *M. tuberculosis*–triggered necrosis involves damage to phagolysosomes in which the bacteria are residing [10]. IFN-γ may promote such damage and facilitate macrophage necrosis. However, no IFN-γ–enhanced damage to the *M. tuberculosis*–containing phagolysosomes was observed (data not shown). We and others have shown that ESX-1 mediates phagosomal rupture [8–10]. ESAT-6 point mutants defective in damaging phagosomes are unable to induce necrosis [10] and are unlikely to cause extracellular trap formation. The elastase inhibitor AAPV, which blocked extracellular trap formation, did not affect damage of phagosomes or necrosis (data not shown; Figure 1*A*). Therefore, extracellular trap formation could be a downstream effect of ESX-1–dependent phagosomal damage and necrosis. Alternatively, phagosomal damage and extracellular trap formation could be independent processes simultaneously induced by ESX-1.

Vogt and Nathan have recently reported that human macrophages cultured in the presence of GM-CSF and 40% serum at a physiological O_2_ level (10% O_2_) survive *M. tuberculosis* infection for several weeks [27]. Key differences between their study and ours included MOIs (0.1–0.2 vs 5–10 in this study), the strains used (Erdman vs H37Rv in this study), and serum percentage (40% vs 10% in this study). With 40% serum, a low IFN-γ level (2.5 U/mL) could still amplify ESX-1–induced necrosis (data not shown). Although we did not determine IFN-γ's effect by using a lower MOI of Erdman, which is less virulent than H37Rv [46], we speculate that the more virulent the infection is, such as one involving a higher MOI and a more virulent *M. tuberculosis* strain, the greater the likelihood that IFN-γ can enhance *M. tuberculosis–*triggered necrosis.

Necrosis induced by *M. tuberculosis* represents a nonresolving inflammation [47]. Necrotic cells release inflammatory cytosolic contents, yet these cells are unable to reduce the *M. tuberculosis* burden. As this necrosis is facilitated by IFN-γ as reported here, *M. tuberculosis*–infected human macrophages are more likely to undergo necrosis when IFN-γ is present, such as during the adaptive immune response against *M. tuberculosis* antigens. Because IFN-γ's potentiating effect is ESX-1 dependent, our work suggests that targeting ESX-1 activity should prevent IFN-γ from inducing human macrophages to undergo a nonprotective necrosis. Patients with active tuberculosis have elevated IFN-γ levels [48–50]. Patients receiving aerosolized IFN-γ show clinical improvement [45]. Thus, improving IFN-γ's efficacy against *M. tuberculosis* might be possible by targeting ESX-1's pronecrotic effect on *M. tuberculosis–*infected macrophages.

## Supplementary Data

Supplementary materials are available at *The Journal of Infectious Diseases* online (http://jid.oxfordjournals.org/). Supplementary materials consist of data provided by the author that are published to benefit the reader. The posted materials are not copyedited. The contents of all supplementary data are the sole responsibility of the authors. Questions or messages regarding errors should be addressed to the author.

Supplementary Data
