# Supplementary material for: Mycobacterium tuberculosis Exploits Human Interferon γ to Stimulate Macrophage Extracellular Trap Formation and Necrosis
Source: J Infect Dis. 2013 Mar 8;208(1):109–19. doi: 10.1093/infdis/jit097 (PMC3666134; doi:10.1093/infdis/jit097)
Supplement: Supplementary Data [file supp_208_1_109__index.html]

Mycobacterium tuberculosis Exploits Human Interferon γ to Stimulate Macrophage Extracellular Trap Formation and Necrosis — Mycobacterium tuberculosis Exploits Human Interferon γ to Stimulate Macrophage Extracellular Trap Formation and Necrosis — Supplementary Data 

# *Mycobacterium**tuberculosis* Exploits Human Interferon γ to Stimulate Macrophage Extracellular Trap Formation and Necrosis

## Supplementary Data

Supplementary Data

**Files in this Data Supplement:**

- Supplementary Data - Pdf file
