# Supplementary material for: Mycobacterium tuberculosis Exploits Human Interferon γ to Stimulate Macrophage Extracellular Trap Formation and Necrosis
Source: J Infect Dis. 2013 Mar 8;208(1):109–19. doi: 10.1093/infdis/jit097 (PMC3666134; doi:10.1093/infdis/jit097)

Fig. S1. Phase contrast images of infected macrophages under different culture conditions. (A) Effect of GM-CSF differentiation and IFN-γ activation. Primary human macrophages differentiated with or without GM-CSF in the presence of 10% human serum were activated with 100 U/ml IFN-γ or untreated before infection with sonicated *M. tuberculosis* H37Rv at a MOI of 10. (B) Effect of oxygen levels. Primary human macrophages were differentiated with GM-CSF either at normoxia (~20% O_2) or at physiological oxygen level (10% O_2) and pretreated with 100 U/ml IFN-γ before infection with non-sonicated *M. tuberculosis* at a MOI of 5. Scale bar, 100 μm.

Fig. S1

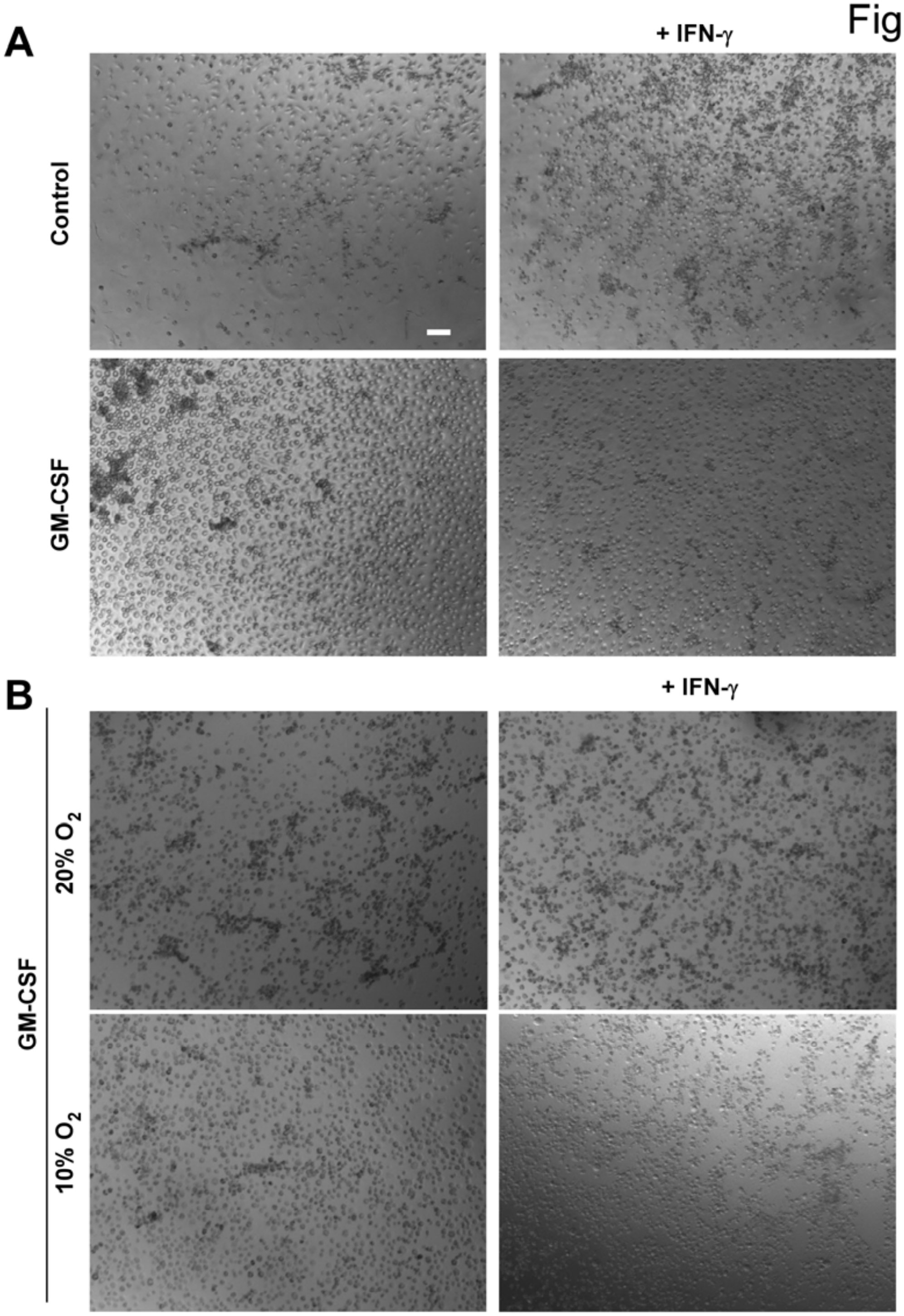

Supplement: Supplementary Data [file supp_jit097_jit097supp.pdf]
